# Transcriptome-based deep learning analysis identifies drug candidates targeting protein synthesis and autophagy for the treatment of muscle wasting disorder

**DOI:** 10.1038/s12276-024-01189-z

**Published:** 2024-04-01

**Authors:** Min Hak Lee, Bada Lee, Se Eun Park, Ga Eul Yang, Seungwoo Cheon, Dae Hoon Lee, Sukyeong Kang, Ye Ji Sun, Yongjin Kim, Dong-sub Jung, Wonwoo Kim, Jihoon Kang, Yi Rang Kim, Jin Woo Choi

**Affiliations:** 1 College of Pharmacy, Kyung Hee University, Seoul, 02447 Republic of Korea; 2 Department of Biomedical and Pharmaceutical Sciences, Kyung Hee University, Seoul, 02447 Republic of Korea; 3 Department of Pharmacology, Institute of Regulatory Innovation Through Science, Kyung Hee University, Seoul, 02447 Republic of Korea; 4 Center for Research and Development, Oncocross Ltd, Seoul, 04168 Republic of Korea

**Keywords:** Bioinformatics, Autophagy, Mechanisms of disease

## Abstract

Sarcopenia, the progressive decline in skeletal muscle mass and function, is observed in various conditions, including cancer and aging. The complex molecular biology of sarcopenia has posed challenges for the development of FDA-approved medications, which have mainly focused on dietary supplementation. Targeting a single gene may not be sufficient to address the broad range of processes involved in muscle loss. This study analyzed the gene expression signatures associated with cancer formation and 5-FU chemotherapy-induced muscle wasting. Our findings suggest that dimenhydrinate, a combination of 8-chlorotheophylline and diphenhydramine, is a potential therapeutic for sarcopenia. In vitro experiments demonstrated that dimenhydrinate promotes muscle progenitor cell proliferation through the phosphorylation of Nrf2 by 8-chlorotheophylline and promotes myotube formation through diphenhydramine-induced autophagy. Furthermore, in various in vivo sarcopenia models, dimenhydrinate induced rapid muscle tissue regeneration. It improved muscle regeneration in animals with Duchenne muscular dystrophy (DMD) and facilitated muscle and fat recovery in animals with chemotherapy-induced sarcopenia. As an FDA-approved drug, dimenhydrinate could be applied for sarcopenia treatment after a relatively short development period, providing hope for individuals suffering from this debilitating condition.

## FDA-approved dimenhydrinate boosts muscle regeneration in sarcopenia models

Scientists used artificial intelligence to find a possible cure for sarcopenia, a disease marked by muscle loss and decreased function. The group, headed by Dr. Jin Woo Choi, used AI to study gene expression patterns in muscle tissues from healthy people and sarcopenia patients. They found a medicine named dimenhydrinate (DH) as a possible cure. The group then experimented with DH on lab-grown muscle cells and mice, discovering that it encouraged muscle cell growth and repair. The scientists concluded that DH could be a hopeful cure for sarcopenia. However, more studies are required to validate these results and to comprehend how DH functions at a molecular level.

This summary was initially drafted using artificial intelligence, then revised and fact-checked by the author.

## Introduction

A hallmark of sarcopenia is muscle weakness resulting from an involuntary loss of muscle mass and the associated functions^1^. However, despite the World Health Organization’s classification of sarcopenia as a distinct disease, the pathogenic mechanisms of sarcopenia are not clearly defined given its interplay with many physiological conditions^2,3^.

Primary sarcopenia, associated with aging, is the most prevalent type of sarcopenia and involves quantitative and qualitative declines in skeletal muscle structure and function^4^. Secondary sarcopenia, on the other hand, is strongly associated with various diseases, including cancer, amyotrophic lateral sclerosis (ALS), Duchenne muscular dystrophy (DMD), and other degenerative diseases. Sarcopenia in cancer patients affects physical function and overall quality of life^5^. Multiple factors, such as the side effects of chemotherapeutic agents, loss of appetite, metabolic changes, increased inflammatory responses, and altered catabolic pathways, contribute to sarcopenia in these patients^6–8^. Furthermore, patients with advanced cancer often experience spontaneous weight, fat, or muscle volume loss, primarily caused by cancer cachexia. Studies have also linked sarcopenia to decreased life expectancy and increased relapse rates within 5 years post surgery in cancer patients^9^. Additionally, sarcopenia is an important issue for patients with rare muscular diseases, such as amyotrophic lateral sclerosis (ALS), DMD, and multiple sclerosis (MS). The exact causal relationship between these diseases and sarcopenia is still unclear; however, it is evident that sarcopenia significantly impacts patient mortality and prognosis^10,11^.

Characteristically, sarcopenic muscles exhibit a reduced myofiber number, particularly Type II fibers, and hypotrophy^12^. Adipose tissue accumulation and fibrotic tissue infiltration are common as the condition progresses^13^. Despite being dormant, a diminished population of muscle satellite cells (MuSCs) can be activated and proliferate into myoblasts, aiding muscle regeneration^14–16^. In light of this, manipulating and eliciting appropriate proliferation and differentiation processes may be promising strategies for investigating therapeutic interventions for sarcopenia. Given the abundant presence of mitochondria in skeletal muscle, which serve as the primary producers of ATP through oxidative phosphorylation, the generation of reactive oxygen species (ROS) is closely intertwined with various manifestations of muscle pathophysiology^17^. A substantial body of evidence indicates a negative correlation between elevated ROS levels and the regenerative capacity of MuSCs^18–20^, underscoring the importance of mitigating ROS production.

Furthermore, the autophagy signaling pathway is pivotal for preserving skeletal muscle mass and myofiber integrity^21^. Notably, many studies have highlighted the importance of autophagy in the activation and differentiation of MuSCs^22,23^. In addition, disruption of autophagy at different stages, such as initiation, cargo trafficking, or lysosomal fusion, hampers myogenic differentiation and impairs strength recovery following muscle injury^24,25^. Taken together, these findings underscore the importance of autophagy in myogenic differentiation, emphasizing its regulatory influence on myoblast differentiation and fusion.

Sarcopenia is triggered by multiple cellular and molecular factors, including chronic inflammation, protein synthesis and turnover imbalance, mitochondrial dysfunction, and oxidative damage^26^. Oxidative stress leads to the abnormal buildup of inflammatory cytokines, primarily proinflammatory cytokines^17^. This process activates macrophages and neutrophils, promoting phagocytic activity and the release of cytotoxic proteases, proinflammatory cytokines, and ROS^27^. Again, excessive ROS production damages mitochondria, reduces ATP levels, increases protein degradation, and hinders protein synthesis, leading to muscle loss and weakened strength^28^. In addition, muscle protein degradation rates surpass muscle protein synthesis rates during sarcopenia development, gradually leading to severe muscle mass loss^29^. The mammalian target of rapamycin complex 1 (mTORC1), a nutrient sensor, helps maintain muscle homeostasis by integrating signals related to amino acid availability, energy status, and the status of the insulin signaling pathway^30^. Mitochondrial dysfunction, which results in muscle degeneration, has also been emphasized as an important cause of sarcopenia. Insufficient energy generation in mitochondria has been repeatedly reported in animal and human biopsy samples^31^. Despite this knowledge at the molecular level, none of the current therapeutic strategies address the root causes of sarcopenia^32^.

Over the past several decades, the practice of drug repurposing has emerged as a potent strategy for addressing a multitude of human diseases^33^. However, the formidable challenge of developing effective and affordable treatment approaches for complex diseases persists in the absence of comprehensive knowledge regarding the entire drug–target network^34^. Recently, a plethora of advanced computational techniques have been devised that incorporate innovative machine learning (ML) and artificial intelligence (AI) algorithms^35^. These approaches aim to revolutionize the drug development process by providing a more systematic and scientifically sophisticated framework. By leveraging algorithms, researchers can detect structural or network similarities between signatures induced from diverse entities, such as drugs, diseases, proteins, and genes^36^. Given that drug repurposing relies heavily on vast quantities of observed data pertaining to existing drugs and diseases, the exponential growth of publicly available, large-scale machine learning methods has paved the way for state-of-the-art applications in the field of data science^37^.

ML and deep learning algorithms were employed in this study to predict potential drugs that ameliorate muscle loss from transcriptome data derived by chemical and disease conditions. Our findings may hold promise for future clinical applications, potentially offering a new direction for sarcopenia treatment.

## Materials and methods

### Deep learning-aided drug screening

#### Collecting RNA expression data and preprocessing

The drug candidate screening process began with collecting disease- or drug-related transcript expression data. The RNA expression data of muscle tissues from healthy individuals and cancer patients who suffer from sarcopenia were collected from the GSE20571 and GSE34111 datasets obtained from the Gene Expression Omnibus (GEO). The drug-related RNA expression information was obtained using a large-scale public database called Connectivity Map and LINCS cloud 3.0. The preprocessing step consisted of outlier fitting, quantile normalization, and scale standardization and was conducted to ensure consistency between each RNA dataset derived from different batch studies containing the RNA expression datasets.

#### DEG selection

The transcriptome data of each sample was input into the differentially expressed gene (DEG) selection algorithm of RAPTOR AITM using preprocessed RNA data. In this process, DEGs between control and experimental samples were identified by multiple statistical tests.

#### Drug-disease comparison and score ensemble

Combinatorial analysis of disease-derived DEGs and drug-derived DEGs was conducted to score each drug for its potential efficacy on the target disease. Three individual scores were obtained from the enrichment test (ET), similarity test (ST), or contingency test (CT). The ET was used to determine the relative distribution of drug-related or disease-related DEGs to one another in terms of DEG expression levels. The ST was used to determine the similarity of DEG expression patterns between the two groups (drug and disease) from the vectorized DEG expression levels. A larger angle (θ) between the vectors (low similarity) of the two groups indicated a more suitable drug for the target disease. From the CT, statistical contingencies were analyzed by commonly expressed DEGs between the two groups (drug and disease). In other words, the statistical significance of the intersection between highly expressed drug-derived DEGs and disease-derived DEGs with low expression (and vice versa) was measured during the ST. Consequently, the three individual scores calculated from these tests composed the final score of a drug candidate during the score ensemble process. The screened drug candidates with higher scores were hypothetically more effective at restoring normal gene expression patterns in sarcopenia patients.

### Cells and reagents

#### C2C12 and muscle satellite cell subculture

The mouse myoblast cell line C2C12 from the American Type Culture Collection (ATCC, USA) was cultured in Dulbecco’s modified Eagle’s medium (DMEM; Invitrogen Co., USA) supplemented with high glucose, 10% (v/v) fetal bovine serum (FBS; Invitrogen Co., USA) and 1X penicillin/streptomycin (Welgene, Inc., Korea). The cells were cultured and maintained at ~20% confluency at 37 °C and 5% CO_2_ in a humidified incubator. C2C12 cells were treated with 400 µM H_2_O_2_ in the medium for 24 h–6 days to induce oxidative stress^38,39^. Muscle satellite cells isolated from mouse skeletal muscles were incubated and cultured via the same method.

#### C2C12 and muscle satellite cell differentiation

C2C12 cells and mouse muscle satellite cells were seeded at 50–60% confluence 2 days before inducing differentiation. Differentiation was induced by replacing 10% FBS with 2% horse serum (Invitrogen Co., USA) and culturing for 1–11 days^40^. The differentiation media was changed every 2 days.

#### Reagents

Dimenhydrinate (D2396, Sigma-Aldrich, USA), 8-chlorotheophylline (C0293, Tokyo Chemical Industry, Japan), diphenhydramine (D0423, Sigma-Aldrich, USA) and 5-fluorouracil (F6627, Sigma-Aldrich, USA) were dissolved in DMSO (D8418, Sigma-Aldrich, USA) at the highest concentration indicated in the product manuals and stored at −20 °C. H_2_O_2_ (39155-1250, Junsei Chemical, Japan) was diluted to a suitable concentration in distilled water (DW) just before use. Recombinant human TNF-α (210-TA-005/CF; R&D Systems, USA) was diluted to 50 ng/ml in the appropriate buffer according to the manufacturer’s instructions and stored at −70 °C. Cardiotoxin from *Naja pallida* (217503, Sigma-Aldrich, USA) was dissolved in PBS at 1 mg/ml and stored at −20 °C. BaCl_2_ (B0750, Sigma-Aldrich, USA) was diluted to 1.2% (w/v) in DW and stored at −20 °C. For in vivo experiments, chemicals were diluted directly in PBS or DW at the desired concentration.

### MTT assay

To assess cell viability, we used a 3-(4,5-dimethylthiazolyl-2)-2,5-diphenyltetrazolium bromide (MTT) assay^41^. MTT was purchased from Duchefa Biochemie (Netherlands). MTT was dissolved in PBS at 5 mg/ml, sterilized by 0.22 µm filtration, and stored at −20 °C. C2C12 cells were seeded at 50–60% confluence and stabilized for at least 16 h. After 24 h of incubation in 10% (v/v) FBS-DMEM with or without H_2_O_2_ or drug candidates, a 1:10 volume of MTT solution was added to each well, and the mixture was incubated for 4 h at 37 °C in a CO_2_ incubator. After that, the cells were carefully washed with PBS, and 100 µl of DMSO was added to dissolve the formazan crystals. The optical density (OD) values were measured at 620 nm using a microplate reader (Tecan, Switzerland). The experiments were repeated three times independently.

### BrdU incorporation assay

The BrdU incorporation assay was performed according to the manufacturer’s protocol using a BioVision BrdU Cell Proliferation Assay Kit (#K306-1000). Cell monolayers were treated with BrdU and incubated at 37 °C for 1 h to allow for incorporation of DNA. BrdU incorporation was determined using a specific primary antibody and an HRP-conjugated secondary antibody, and the absorbance was measured at 450 nm.

### Cell counting assay

The cells were detached from the culture dish using trypsinization and resuspended in an appropriate volume of media. Subsequently, the cell suspensions were diluted 1:1 with 0.4% trypan blue solution (15250-061; Thermo Fisher Scientific, USA). Ten microliters of mixture were placed in a hemocytometer, and live cells within the four counting areas (1 mm^3^ each) were counted under a microscope. Cell counts were conducted three times per sample for statistical analysis.

### Western blot analysis

Before transferring the proteins to a PVDF membrane, the prepared protein samples were loaded into a 10% acrylamide gel for western blotting electrophoresis and blocked with a 5% skim milk solution. The membrane was incubated in primary antibody diluted with Tris-buffered saline (TBS) supplemented with 0.05% Tween (TBS-T) for 2 h. After three washes with TBS-T, the diluted secondary antibody was incubated for 1 h. Detection was performed after the addition of the luminol mixture (TLP-112.1; Translab, Korea). The following primary antibodies were used for western blotting: ATG5 (sc-515347, Santa Cruz, USA), p21 (sc-6246, Santa Cruz, USA), MyoD (sc-377460, Santa Cruz, USA), myogenin (sc-12732, Santa Cruz, USA), MYH (sc-376157, Santa Cruz, USA), LC3 (2775S, Cell Signaling Technology, USA), mTOR (2972S, Cell Signaling Technology, USA), p-mTOR (2974T, Cell Signaling Technology, USA), eIF2-α (9722S, Cell Signaling Technology, USA), p-eIF2α (9721S, Cell Signaling Technology, USA), Nrf2 (sc-365949, Santa Cruz, USA), p-Nrf2 (PA5-67520, Invitrogen, USA), β-actin (sc-47778, Santa Cruz, USA) and α-tubulin (sc-8035, Santa Cruz, USA). For secondary antibodies, we used horseradish peroxidase (HRP)-conjugated goat antibody against rabbit IgG (PA489724, CusaBio, USA) and HRP-conjugated goat antibody against mouse IgG (PA644737, CusaBio, USA).

### In vivo toxin-induced muscle damage model

All animal experiments were approved by the Institution of Animal Care and Use Committee of Kyung Hee University under permit number KHUASP(SE)-18-101. The animals were housed in a room with a 12 h light cycle and provided free access to water and a standard diet.

#### Cardiotoxin-induced mouse model

To evaluate the effect of the drug candidates on acute muscle damage, 1 µg of cardiotoxin was injected intramuscularly into the gastrocnemius muscle of 8-week-old male C57BL/6 mice^42^. Furthermore, 3 days after injection, PBS or 30 mpk DH was administered intraperitoneally once daily for 11 days (from day 3 to 13). On day 14, the mice were sacrificed, and the TA muscles were dissected. After dissection and two washes in ice-cold PBS, the muscles were embedded in paraffin.

#### BaCl_2_-induced mouse model

To establish a toxin-induced muscle damage model, we used a Hamilton syringe to inject 50 µl of 1.2% BaCl_2_ into the gastrocnemius region. Three days after the injection, DH was administered at a dose of 30 mpk via intraperitoneal (IP) injection. Three days after DH treatment once a day, the mice were sacrificed, and the gastrocnemius muscles were isolated to measure their size. As mentioned earlier, we prepared and dissected the tissue and performed staining accordingly.

#### Muscle fiber diameter quantification

Following the precision sectioning of paraffin-embedded muscle specimens from each distinct experimental cohort to a transverse thickness of 4 μm, a series of four contiguous sections for each mouse sample were meticulously observed under a magnification factor of 100. Subsequent to image acquisition, the diametric dimensions of no fewer than 15 cross-sectional muscle fiber profiles were ascertained within both the damaged regions and unaffected regions within the visual field. This analytical methodology was then systematically employed for evaluating the left and right gastrocnemius muscles.

#### Locomotor assessment

The pole test procedure was similar to the method established by Ogawa et al.^43^. In summary, the animals were placed near the top of a rough-surfaced wood pole measuring 1 cm in diameter and 50 cm in height, with their heads oriented upward. The elapsed time until they executed a full downward turn, referred to as a “T-turn”, and descended to the floor was carefully recorded. A maximum allowable time of 180 s was set for this assessment.

The four-limb hanging test was performed according to the method established by Aartsma-Rus et al.^44^. The experimental procedure involved placing the mouse on an inverted metal grid positioned above a bedding-filled cage. The test concluded either when the mouse achieved a maximum hanging time of 600 s or, in cases of earlier falls, after three test sessions. Subsequent analysis utilized the maximum hanging time observed across all trials.

### Primary cell isolation from wild-type and MDX mice

Needle puncture was applied to the tibialis anterior (TA) and gastrocnemius muscles using a syringe to induce muscle damage and MuSC activation in 8-week-old wild-type or MDX mice. The mice were euthanized 3 days postpuncture. The subsequent extraction of primary myoblasts from the acquired muscles involved mincing the muscle tissue and incubating it in DMEM supplemented with 20 mg/ml collagenase type II for 2 h. The homogenate was then subjected to filtration via a 30 μm strainer and centrifugation. The resulting cells were seeded into a culture plate to promote their proliferation.

### Cancer formation and 5-FU chemotherapy-induced muscle wasting model experiments

#### Carcinoma cell line-allograft mouse model

As shown in Fig. 2, the flanks of 7-week-old male CD2F1 mice were injected with C26 carcinoma cells. After the allograft volume was >100 mm^3^, the mice were treated with 20 mpk of DH. All of the treatments were administered daily through oral gavage for 19 days. Mice were fed an AIN-93 M-based diet ad libitum.

As shown in Fig. 8, an allograft model using the mouse colon carcinoma cell line CT26 was established to assess the effect of drug candidates on cancer formation and 5-FU chemotherapy-induced muscle wasting. 8-week-old male BALB/c mice were anesthetized with isoflurane, and 1 × 10^6^ CT26 cells per mouse were injected subcutaneously (day 0). From days 8–18, 5-FU was administered thrice weekly through intraperitoneal injection at 50 mpk. The drug candidates were orally administered at a designated concentration daily from days 0–18. All mice had free access to the AIN-93 M-based diet. Fat and muscle volume (mm^3^) were measured using magnetic resonance imaging on days 1, 8, 15, and 18. Oncocross Co. (Seoul, Korea) and the Research & Business Development Center of Seoul Asan Hospital (Seoul, Korea) approved the study.

#### RNA sequencing

RNA from the gastrocnemius was isolated and sent to Novogene for sequencing. Quality control of the RNA was performed by measuring the RNA integrity number (RIN) via an Agilent Bioanalyzer. All samples exceeded the minimal acceptable RIN (>4.6). After that, the resulting cDNA libraries were constructed and sequenced on an Illumina NovaSeq platform to generate 20 million 150 bp paired-end reads per sample.

#### Quality control

Read quality control was performed using FastQC, and the results were aggregated using the MultiQC tool. All the samples exhibited high read quality, with PHRED scores consistently above 25 across read positions and <50% nonunique sequences for all the samples. Further quality control was performed by visualizing and quantifying the library size and log CPMs. Library sizes showed minimal variance, with most samples exhibiting nearly the targeted 20 million reads. All the samples showed a consistent gene count per million reads (CPM) distribution. The overall technical quality of the experiment was high. Before model construction, genes that did not have at least five counts in at least eight samples were filtered out. The remaining 10,547 genes passed the filtering criteria.

#### Alignment and annotation

The reads were aligned to the *Mus musculus* GRCm39 genome build 103 using the subread software package (v2.0.1). Annotation of genomic features was performed using the featureCounts program, which is part of the Subread software package. The *Mus musculus* GRCm39 build 103 GTF file was used as a reference for annotation. The genomic fasta and gross total fragment (GTF) references were obtained from the ENSEMBL genome browser ftp.

#### Weighted gene coexpression network analysis (WGCNA)

The WGCNA algorithm was used as an alternative method to determine drug-induced changes in the transcriptome by assessing coexpression patterns across all samples. In addition, this technique allows for correlation between phenotypic outcomes and gene expression patterns to be determined. This allows for hypothesis generation regarding genes or networks of genes that could be regulated by drug treatments and/or that drive phenotypic responses.

#### Magnetic resonance imaging (MRI) of muscle and fat

A single prescan was performed prior to tumor xenografting, and individuals meeting the criteria for cachexia (tumor size >100 mm³) were selected. Subsequently, MRI scans were conducted weekly, totaling three sessions. The field strength was set to 9.4 tesla MRI scanner (Agilent, Inc.), and sequence and imaging parameters were measured via fast spin echo T1-weighted imaging (TR/TE = 1100/10.75 ms, field of view = 30 × 30 mm, slice thickness = 1 mm and matrix size = 128 × 128). The field of view was set to cover the liver and kidney of the animal, with respiration gating for in vivo imaging. On MR images, the area of fat/muscle was measured by adjusting the threshold for the range of signal intensity of fat and muscles using AsanJ-TM, which is a modified version of ImageJ (https://aim-aicro.com/software/morphometry). The regions of interest (ROIs) for fat and muscle areas were automatically determined based on the signal values corresponding to fat and muscle in the images using the AsanJ program algorithm. ROIs for fat and muscle were selected for each slice of the captured images. The total quantities of fat and muscle were calculated using the following formula: V = Th × S (V: total volume of muscle or fat, Th: Thickness, S: sum of ROIs).

### Tissue preparation

At the end of the behavioral assessment, we immersed the samples in a 4% paraformaldehyde solution in PBS (4% PFA) at 4 °C overnight to analyze the isolated muscle tissue. Next, we used a tissue processor to perform paraffinization. Once the tissues were paraffinized, we used a tissue embedder to mold them, and then the issues were subsequently sectioned into 4 µm-thick sections using a microtome.

### Hematoxylin and eosin staining

The slides with tissue sections were immersed in xylene or a substitute for deparaffinization. This step was followed by rehydration using decreasing concentrations of ethanol. The slides were rinsed with distilled water and immersed in Harris hematoxylin solution, after which the nuclei of the tissue sections were stained. After a specific duration, the slides were rinsed with running tap water and immersed in eosin Y solution for a fixed period. Following eosin staining, the slides were rinsed with distilled water to remove excess dye. Subsequently, the slides were dehydrated by transferring them through increasing concentrations of ethanol and xylene. Finally, Canada balsam was applied to the tissue sections, and a glass coverslip was placed over the sections, ensuring that there were no air bubbles.

### Immunohistochemistry (IHC) staining

The tissue sections were deparaffinized and rehydrated following established procedures. Subsequently, heat-induced antigen retrieval was achieved by immersing the sections in sodium citrate buffer (10 mM, 0.05% Tween 20, pH 6.0) for 10 min. After cooling, the sections were thoroughly washed with TBS-T (0.05% Tween 20). To prevent nonspecific binding, the sections were blocked with 1% bovine serum albumin (BSA100, LPS solution, Korea) in TBS-T for 1 h at room temperature and then treated with 0.3% H_2_O_2_ in TBS for HRP detection. The same primary antibodies used in the western blot experiments were employed and the proteins were diluted according to the manufacturer’s recommendations. The sections were incubated overnight at 4 °C with their respective primary antibodies. Next, the slides were washed with TBS-T and subjected to a 15 min incubation with the secondary antibody, which is part of the ABC complex kit, at 37 °C. Subsequently, an ABC complex kit (Wuhan Boster Biological Technology, Ltd., Wuhan, China) was used. The development of color and visualization were achieved by adding 3,3‑diaminobenzidine. Finally, the tissue sections were lightly counterstained with hematoxylin, dehydrated, cleared, and coverslipped. The slides were subsequently imaged using a light microscope.

### Immunofluorescence (IF) staining

The cells attached to a coverslip were then moved to a glass slide for staining. After washing with PBS three times for 5 min, the cells were blocked with 5% BSA in PBS for 30 min, followed by permeabilization with 0.2% Triton X-100 in PBS. The primary antibody was diluted with 1% BSA in PBS, applied to the sections/cells, and incubated at room temperature for 1 h. After three washes with PBS-T (0.1% tween in PBS) for 5 min, the secondary antibody was diluted with 1% BSA in PBS and incubated in a dark room for 1 h. Mounting with VECTASHILD antifade mounting medium and DAPI was used for nucleus counterstaining.

### Statistical analyses

The *p* values are presented as the mean ± SEM, and the data were statistically analyzed using Student’s *t* test or ANOVA, where appropriate. A *p* value < 0.05 indicated statistical significance.

## Results

### An artificial intelligence (AI)-aided analysis revealed that dimenhydrinate has the potential to promote muscle regeneration

**Fig. 1:**
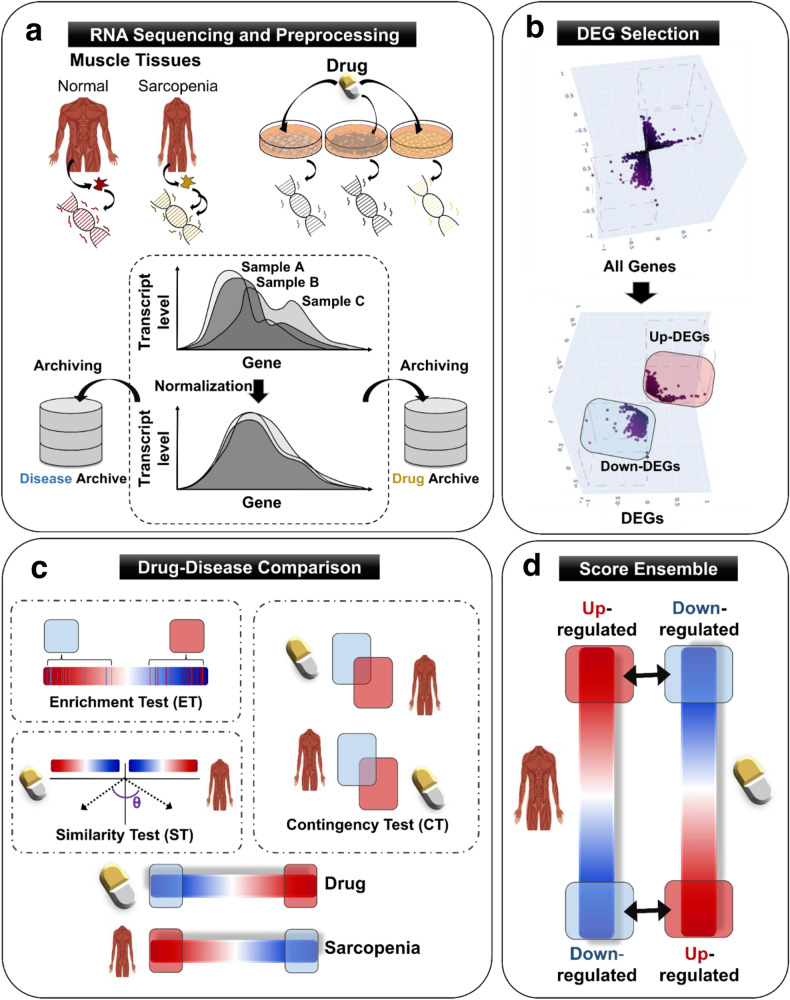
AI-aided analysis for screening chemical candidates. **a** Scheme of RNA sequencing and preprocessing. Transcript expression information from muscle tissues isolated from healthy individuals and patients afflicted with sarcopenia was processed and analyzed. The objective of this study was to identify potential drug candidates capable of rebalancing patients’ aberrant gene expression profiles toward a normal state. **b** Differentially expressed gene (DEG) selection: RNA expression data were collected from the muscle tissues of healthy hosts and cancer patients suffering from sarcopenia. **c** Scheme showing the drug-disease comparison. Comprehensive analysis of disease-associated DEGs in two different cachexia cohorts. Drug-induced DEGs were used to evaluate the efficacy of each drug against the target disease. **d** Schematic of the score ensemble: three distinct scores, derived from the enrichment test (ET), similarity test (ST), and contingency test (CT), were combined for further analysis.

Given the absence of available therapeutic interventions for sarcopenia, we tried to identify potential drug candidates for effective and efficient treatment using an artificial intelligence (AI)-aided analysis method (Fig. 1). We constructed a transcript database containing RNA-seq data from diseased and drug-treated conditions (Fig. 1a). This process followed the comparison of gene expression patterns between pathological and drug-treated conditions conducted by our previously introduced machine learning platform^35^ (Fig. 1b). Briefly, using the random forest and neural network programs, a common expression gene set from a particular tissue was isolated and removed. The different scales among experiments were normalized (Fig. 1c). Finally, the reverse correlation score was predicted between the candidate drug and the targeted disease. Therefore, the system predicted potential drugs that can ameliorate disease and restore normal conditions (Fig. 1d).

**Fig. 2:**
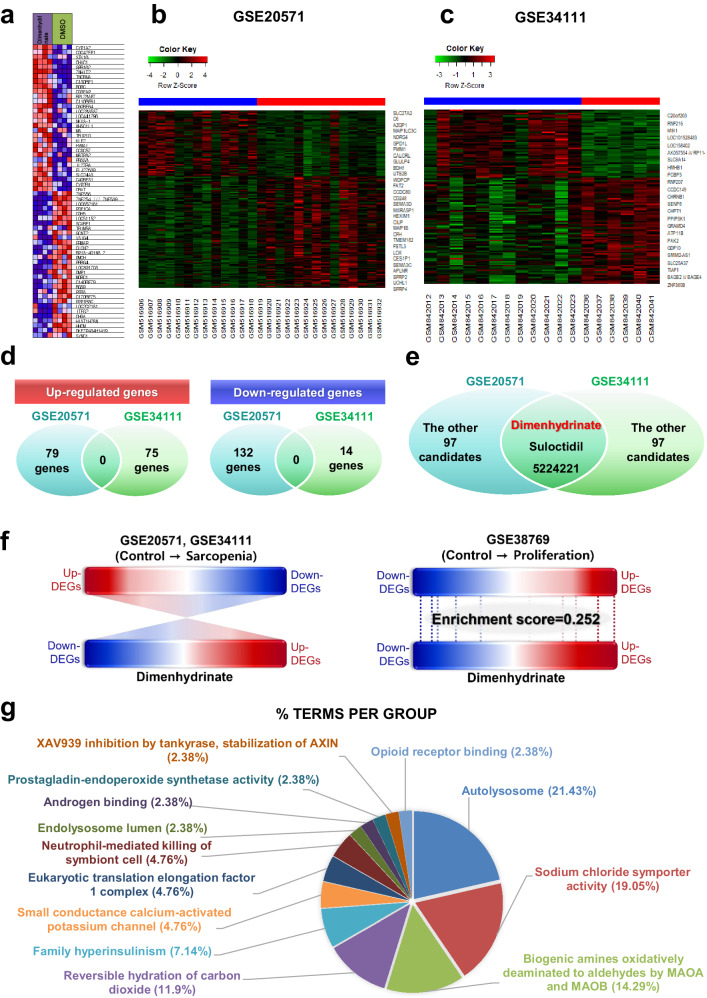
AI-aided analysis for chemical screening. **a** Heatmap depicting the differential gene expression patterns upon DH treatment in comparison to DMSO treatment across three distinct cell lines (HL60, MCF7, and PC3), as elucidated through c-MAP data analysis. **b** Heatmap representation of DH utilizing the GSE20571 dataset. **c** Heatmap representation of DH employing the GSE34111 dataset. **d** Venn diagram illustrating the results of gene analysis using the GEO database and AI-assisted drug analysis. Although there was a lack of shared genes targeted by DH in the two GEO datasets, there were common candidate drugs. **e** Venn diagram depicting the enrichment scoring scheme related to the proliferation effects of DH in the GEO datasets. **f** An illustrative schematic presenting a comparative analysis of the DEG patterns obtained from the GEO datasets (GSE20571 and GSE34111), highlighting the sarcopenia pattern and the DEG pattern resulting from DH treatment (left section). Genes appearing in red were upregulated, and those appearing in blue were downregulated. Schematic diagram presenting the DEG pattern derived from the GEO dataset (GSE38769) with the DEG pattern induced by DH, elucidating the proliferation pattern of human muscle progenitor cells (right section). Genes appearing in red were upregulated, and those appearing in blue were downregulated. Both gene expression patterns had an enrichment score of 0.252. **g** Circular graph illustrating the results of the Gene Ontology (GO) pathway analysis of the DEGs related to DH. DH dimenhydrinate.

For drug candidate screening analysis, utilizing the cohorts from both GEO cancer cachexia datasets GSE20571 and GSE34111 for (Supplementary Table 1a), we performed an in-depth examination of the gene expression patterns observed within these cohorts, as well as the gene expression patterns induced by dimenhydrinate (DH) (Fig. 2a). Subsequently, we identified a set of genes that exhibited significant upregulation or downregulation compared with those in the control group (Fig. 2b from GSE20571 and 2c from GSE34111). We individually screened 100 potential drug candidates (see Supplementary Table 2) and identified a subset of chemicals that emerged as putative drug candidates shared across the GEO dataset cohorts. Notably, upon analyzing the differentially expressed genes (DEGs) obtained from both cohorts, no overlapping genes were observed in either the upregulated or downregulated DEG groups (Fig. 2d). However, utilizing AI analysis, a search for compounds capable of reversing the gene expression patterns (from sarcopenia to a control-like state) yielded three common candidates between the two cohorts (Fig. 2e). Intriguingly, these compounds were shown to induce global changes in gene expression rather than targeting specific genes. These findings suggested that these compounds can dynamically modulate the overall gene expression pattern, thereby causing state transitions. From the three identified candidate compounds, any drugs with a prior history of usage as anticancer agents, antibiotics, or hormones and encountered clinical trial failures or unsuccessful development were excluded from the selection process. Consequently, DH was selected for further investigation (Fig. 2e).

A hypothesis was formulated suggesting that DH can enhance muscle regeneration in individuals with sarcopenia by facilitating the proliferation of MuSCs. Upon comparing the gene expression patterns of control mice with those of model mice, the gene expression patterns were relatively the opposite of each other (left panel in Fig. 2f). To validate whether DH could enhance muscle regeneration in individuals with sarcopenia by facilitating the proliferation of MuSCs, the gene expression patterns of proliferating muscle progenitor cells (GSE38769; see Supplementary Table 1b) and those of cells treated with DH were compared. A significant similarity between the two patterns was discovered (lower panel in Fig. 2f), suggesting that DH induces MuSC proliferation. Interestingly, these in silico Gene Ontology (GO) results suggested that autophagy was the most affected GO term category (Fig. 2g). The detailed descriptions of the samples from each GSE dataset are provided in Supplementary Table 1.

### Dimenhydrinate alters muscle cell metabolism in response to cancer formation and 5-FU chemotherapy-induced muscle wasting

**Fig. 3:**
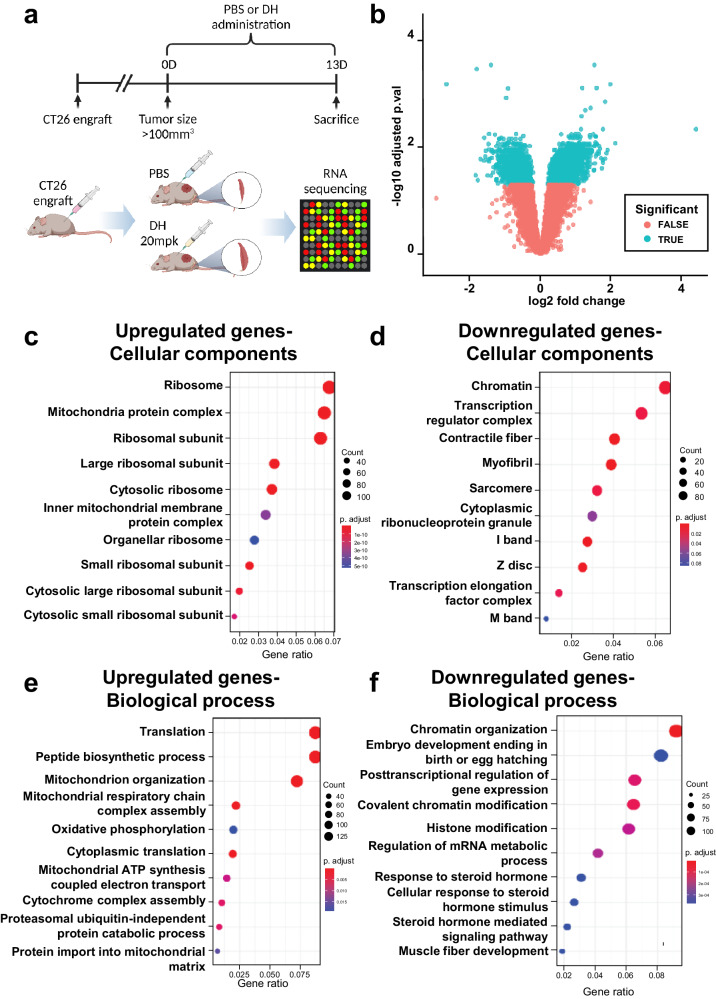
RNA sequencing data for cancer formation and 5-FU chemotherapy-induced mouse tissues. **a** Scheme for analyzing RNA sequencing differences between PBS and DH groups in a CT26-engrafted mouse model. **b** A Volcano plot depicting differential gene expression between vehicle control mice and mice treated with DH. **c**–**f** GO enrichment analysis of the significantly differentially expressed genes in DH-treated tumor-bearing mice versus vehicle-treated nontumor-bearing mice. **c** GO cellular component and **d** biological process pathways associated with upregulated genes. **e** GO cellular component and **f** biological process pathways associated with downregulated genes. DH dimenhydrinate.

An allograft mouse model was established to understand the molecular mechanisms underlying muscle mass preservation by DH using mouse colon cancer cell line CT26 and colon cancer cell lines. CT26 cells were injected into the flanks of CD2F1 mice. When the tumor volume was 100 mm^3^, DH treatment was initiated. After treatment for 13 days, the gastrocnemius muscles were harvested, and RNA was extracted. Then, RNA sequencing was carried out to profile the transcriptomic responses to treatment (Fig. 3a). The establishment and growth of CT26 tumors drove robust changes in skeletal muscle gene expression. When the variation in all the samples was partitioned using a multidimensional scaling technique, there was clear and distinct discrimination between the tumor-bearing and control groups across the first dimension (Supplementary Fig. 1a, b). When assessing differential gene expression in control versus tumor-bearing mouse muscle samples using a full factorial model, 3233 genes were significantly upregulated, and 3549 genes were significantly downregulated. GO enrichment analyses were conducted to identify the enriched cellular components (CCs) and biological processes (BPs) in the DEGs, and the results were slightly different from those above. The significantly upregulated genes were enriched in BPs related to autophagy, phagosome formation (Supplementary Fig. 1d), and CCs including proteasome subunits (Supplementary Fig. 1c). The downregulated genes were enriched in BPs (Supplementary Fig. 1f) and CCs (Supplementary Fig. 1e) related to the extracellular matrix, collagen-related processes, and cellular adhesion.

A volcano plot was generated to investigate the changes in muscle cell metabolism between the DH-treated and control groups. Compared to the vehicle control, DH treatment led to a significant increase in the expression of 1891 genes and a decrease in the expression of 1438 genes (Fig. 3b). Analysis of the cellular components and biological processes associated with the upregulated and downregulated genes revealed that components related to ribosomes were predominantly enriched in the upregulated genes (Fig. 3c). In contrast, muscle-related factors (contractile fibers, myofibrils, sarcomeres) were enriched in the downregulated genes (Fig. 3d). Furthermore, genes involved in biological processes related to muscle synthesis and regeneration, such as translation and peptide biosynthetic processes, were upregulated (Fig. 3e). Finally, we observed a decrease in the expression of genes involved in the overall metabolism of muscle cells, such as genes related to chromatin organization, posttranscriptional regulation of gene expression, and histone modification (Fig. 3f). In summary, DH exerts a protective effect on muscle mass through a variety of molecular mechanisms. It promotes muscle synthesis and ribosomal activities and downregulates muscle-specific factors and genes related to general muscle cell metabolism. These findings provide new insights into how DH combats muscle atrophy at the genetic level.

### 8-Chlorotheophyllin in dimenhydrinate increases cell proliferation

**Fig. 4:**
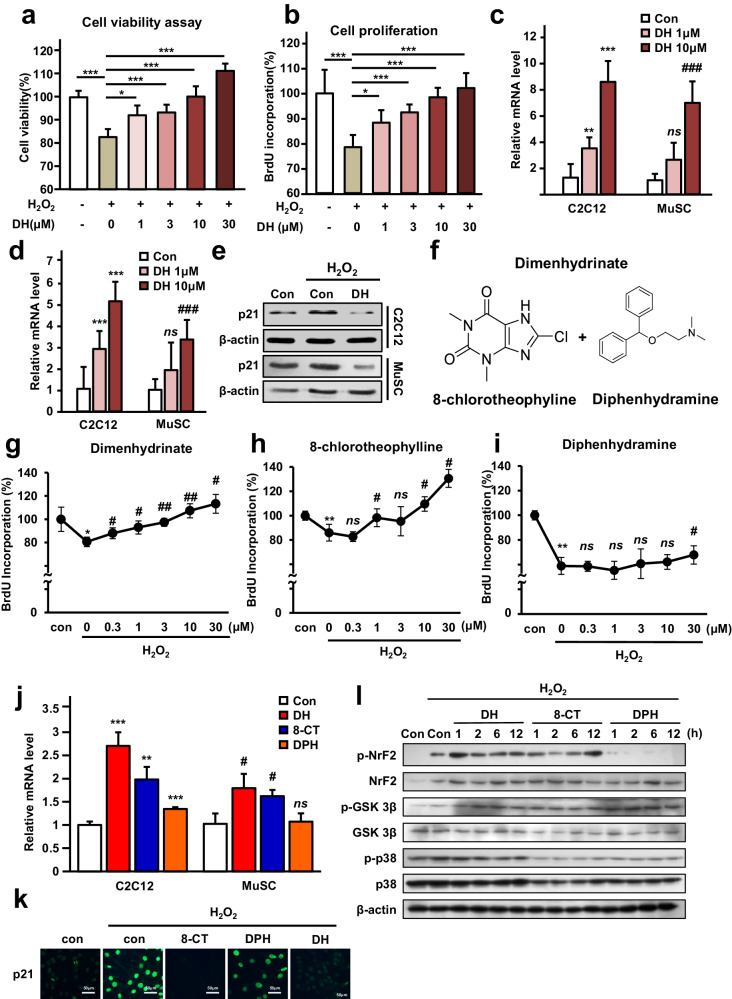
8-Chlorotheophylline in dimenhydrinate promotes muscle cell proliferation by inducing an antioxidative stress mechanism. **a** Cell viability assay (MTT assay) demonstrating the dose-dependent effects of DH under ROS-inducing conditions. **b** BrdU incorporation assay demonstrating dose-dependent effects of DH under ROS-inducing conditions. **c** Relative mRNA expression of cyclin D1 following DH treatment in C2C12 cells and MuSCs. **d** Relative mRNA expression of cyclin E1 following DH treatment in C2C12 cells and MuSCs. **e** Immunoblotting of p21 following a 24 h treatment with or without H_2_O_2_ or DH in both C2C12 cells and MuSCs. **f** Structural depiction of 8-CT and DPH. **g** BrdU incorporation assay for DH. **h** BrdU incorporation assay for 8-CT. **i** BrdU incorporation assay for DPH. **j** Relative mRNA expression of cyclin D1 in response to 8-CT, DPH, and DH treatment in C2C12 cells and MuSCs. **k** Immunofluorescence staining of p21 in response to the three chemicals, as shown in (**j**) (only in C2C12 cells). Scale bars = 50 μm. **l** Immunoblotting in response to the three chemicals as shown in (**j**) under ROS-inducing conditions. For (**a**, **b**, **e**, **g**–**i**, **k**, **l**), H_2_O_2_ (400 μM) was administered with or without chemicals for 24 h. For (**e**, **j**–**l**), all chemicals were administered at a concentration of 10 μM. In (**c**, **d**, **j**), # indicates that the *p* value is significant compared to the MuSC control. In (**g**–**i**), # indicates that the *p* value is significant compared to the H_2_O_2_-treated control. ns not significant, *p < 0.05, **p < 0.01, ***p < 0.005, ^#^*p* < 0.05, ^##^*p* < 0.01, ^###^*p* < 0.005. Error bars: standard deviation (SD). DH dimenhydrinate, 8-CT 8-chlorotheophylline, DPH diphenhydramine, MuSC muscle satellite cell.

A cell proliferation assay with C2C12 myoblast cells was used to investigate the myogenic potential of DH. As investigations involving both in vitro and in vivo approaches to study the link between muscle wasting and oxidative stress in cachexia conditions are actively progressing^45–48^, we chose to induce oxidative stress and mimic cachexia conditions by treating cells with H_2_O_2_. Various DH concentrations were applied to the cells, and H_2_O_2_ was concurrently administered. After 24 h of incubation, the cell viability was found to increase in a dose-dependent manner (Fig. 4a). This pattern was also consistently detected in normal muscle satellite cells (MuSCs), in concordance with the cell count results (Supplementary Fig. 2a). In addition, cell proliferation and viability were increased at various doses, as determined by the BrdU test (Fig. 4b). The levels of cyclin D1 (Fig. 4c) and cyclin E1 (Fig. 4d), cell cycle proteins used as markers of cell proliferation, were elevated in a dose-dependent manner. p21 regulates cell cycle progression by acting as a cyclin-dependent kinase (CDK) inhibitor that can temporarily halt cell division. Upon administering DH under conditions of cell division suppression induced by H_2_O_2_, a reduction in the p21 level was noted (Fig. 4e). These findings were also similar to those observed in normal mouse-derived MuSCs (Fig. 4c–e). Ultimately, we concluded that DH mitigates muscle cell death and regulates the cell cycle, thus promoting proliferation.

DH is a mixture of two drugs, 8-chlorotheophyllin (8-CT) and diphenhydramine (DPH) (Fig. 4f). To determine which chemical is responsible for inducing the proliferation and consequent regenerative effect on muscle cells by DH, cells under the same conditions were treated with either 8-CT or DPH. Interestingly, according to the results of the BrdU (bromodeoxyuridine) incorporation assay, 8-CT (Fig. 4h), but not DPH (Fig. 4i), exhibited a dose-dependent pattern similar to that of DH (Fig. 4g). Comparable outcomes were evident in a cell counting assay employing normal MuSCs. Specifically, an increase in the cell count was detected solely in the cells subjected to concurrent DH and 8-CT treatment following exposure to H_2_O_2_ (Supplementary Fig. 2b). In line with these findings, cell cycle marker measurements also revealed significant changes in cyclin D1 in the presence of 8-CT, and these trends were consistent with those observed in MuSCs (Fig. 4j). In addition, when p21 cells were subjected to immunofluorescence under the same conditions, a decrease in the number of cells treated with 8-CT was confirmed (Fig. 4k). Therefore, we concluded that DH promotes the survival and division of muscle cells via 8-CT. It is well known that oxidative stress often impedes muscle cell proliferation. Additionally, many antioxidative responses occur through the phosphorylation-induced transcription of nuclear factor erythroid 2-related Factor 2 (Nrf2), which serves as a defensive mechanism against oxidative stress^49^. We postulated that the antioxidative mechanism of the 8-CT component of DH against H_2_O_2_ could be associated with the Nrf2 pathway. This pathway was analyzed in cells treated with H_2_O_2_ to verify whether the proliferation of DH cells is contingent on the Nrf2 pathway. As a result, in contrast to the relative stability of p38 mitogen-activated protein kinase (p38-MAPK)^50^ and glycogen synthase kinase-3 beta (GSK3β)^51,52^, which are known as ROS-associated signaling molecules, among the DH, 8-CT, and DPH-treated groups, an increase in p-Nrf2 levels was observed in both the DH and 8-CT assays. However, such a trend was not evident in the DPH assay (Fig. 4l). Therefore, DH exerts its proliferative effects through 8-CT, which triggers the antioxidative response that drives this process.

Given that TNF-α is another main stimulator of muscle wasting, we conducted an assessment to ascertain whether DH could replicate similar effects in an in vitro cachexia model induced by TNF-α. TNF-α slightly induced the proliferation of C2C12 cells (Supplementary Fig. 3a), consistent with prior observations that demonstrated the stimulation of proliferation in primary rat myoblasts^53^. Remarkably, DH in combination with TNF-α strongly induced C2C12 cell proliferation in a dose-dependent manner (Supplementary Fig. 3a). Interestingly, TNF-α, a cyclin-dependent kinase inhibitor, also increased the protein level of p21 (Supplementary Fig. 3b), indicating that TNF-α has complex effects on myogenic cell proliferation and cell cycle regulation. In line with our findings under conditions of elevated ROS, DH mitigated the induction of p21 (Fig. 4e and Supplementary Fig. 3b). In our findings, TNF-α led to an increase in the mRNA levels of cyclin A1 (*CCNA1*), cyclin E1 (*CCNE1*), and cyclin D1 (*CCND1*) (Supplementary Fig. 3c–e). Moreover, 8-CT and DH substantially amplified the expression of all three cyclins at the mRNA level (Supplementary Fig. 3c–e), as did the MuSC marker *PAX7* (Supplementary Fig. 3f). Importantly, these findings align with the findings of individual 8-CT or DH treatment (Fig. 4j). Despite the negative effect of TNF-α on muscle differentiation, both DH and DPH were able to increase the mRNA expression of the differentiation markers *MYOD* and *MYOG* in C2C12 cells (Supplementary Fig. 3f). In concurrence with the results obtained under ROS conditions, DH successfully promoted the proliferation and differentiation of myogenic cells under TNF-α-induced stress conditions.

### Diphenhydramine, a component of dimenhydrinate, enhances muscle differentiation via the autophagy pathway

**Fig. 5:**
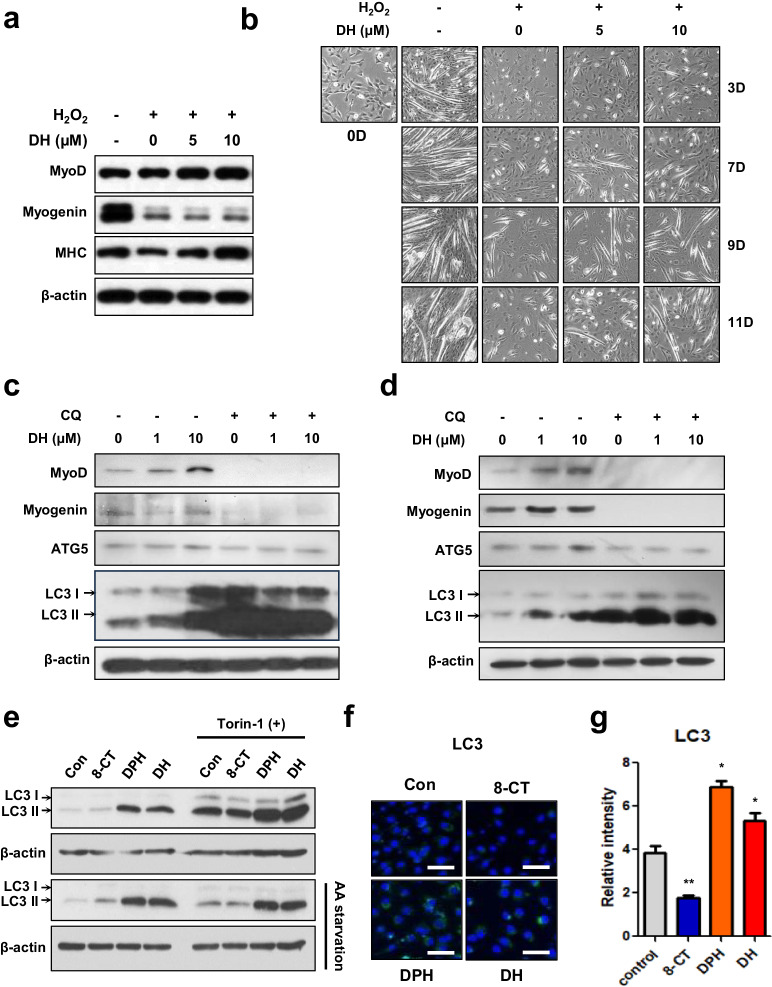
Diphenhydramine in dimenhydrinate promotes muscle differentiation by inducing autophagy. **a** Immunoblotting for MyoD, myogenin, and MHC, which are muscle differentiation markers, during the 7-day differentiation of C2C12 cells. **b** Representative microscopy images of C2C12 cell differentiation following treatment with varying concentrations of DH at different time points. **c** Immunoblotting for MyoD, myogenin, ATG5 and LC3. Chloroquine (CQ; 30 μM, 24 h) was used as an autophagy inhibitor. **d** Immunoblotting for mTOR-independent LC3 via dimenhydrinate after 1 day of differentiation with or without CQ or DH. **e**–**g** The treatment concentrations for 8-CT, DPH, and DH were uniformly set at 10 μM. **e** Confirmation of LC3 induction by target chemicals with or without amino acids. Torin-1 (5 μM, 6 h) was utilized as an mTOR pathway inhibitor. **f** Immunofluorescence staining image of LC3 after individual treatment with the three chemicals. Scale bars = 50 μm. **g** Relative intensity of LC3 in C2C12 cells after treatment with the three chemicals. The mean fluorescence intensity was measured using ImageJ. ns not significant, *p < 0.05, **p < 0.01, ***p < 0.005. Error bars: standard deviation (SD). DH dimenhydrinate, 8-CT 8-chlorotheophylline, DPH diphenhydramine.

To evaluate the potential of DH to enhance myogenic differentiation, C2C12 cells were treated with DH and its components under differentiation conditions. In H_2_O_2_-treated C2C12 cells_,_ stress impeded myogenic differentiation; however, DH upregulated muscle differentiation markers in treated C2C12 cells (Fig. 5a), suggesting that DH can promote myogenesis. The enhancement of these signals during muscle differentiation manifests phenotypically as fiber formation. When DH was administered to C2C12 cells in a differentiation-inhibiting environment caused by H_2_O_2_, a significant increase in the formation of fibers was observed. This effect appeared to be both date- and time-dependent, providing tangible evidence of the compound’s potential to counteract differentiation inhibition (Fig. 5b). Furthermore, in an experiment employing MuSCs, DH demonstrated a notable capacity to effectively stimulate the mRNA expression of myogenic markers under oxidative stress conditions (Supplementary Fig. 2c).

Harsh conditions, such as cachexia, in which nutrients are insufficient to rapidly metabolize unnecessary proteins and other byproducts, can increase the autophagy of muscle-related proteins^54^. Therefore, regulating autophagy is essential for muscle regeneration, growth, and differentiation. In our previous experiments, RNA sequencing (RNA-seq) of CT26-engrafted mice revealed that 5-FU chemotherapy-treated muscle underwent changes in cell metabolism (Supplementary Fig. 1a, b). Notably, in the muscles affected by cancer cachexia, we noted the upregulation of genes related to autophagy, such as those involved in autophagosomes and macroautophagy (Supplementary Fig. 1c, d). To determine whether DH indeed triggers autophagy to facilitate muscle synthesis and differentiation, DH was administered to cells in the presence or absence of amino acids. To determine whether the muscle differentiation ability of DH can be attributed to enhanced autophagy mediated by the microtubule-associated protein 1A/1B-light chain 3 (LC3), the cells were treated with chloroquine (CQ), an autophagy inhibitor^55,56^ and DH, followed by an evaluation of differentiation markers, myogenic differentiation 1 (MyoD) and myogenin, and autophagy-related markers. The results revealed that muscle differentiation induced by DH was diminished in the presence of CQ, underscoring the suggestion that DH promotes muscle differentiation via the autophagy pathway (Fig. 5c). We also noted an increase in both LC3 I and II levels when DH was administered alone, indicating the activation of autophagy. However, when we applied CQ to inhibit autophagy, which led to the accumulation of LC3 II, we observed that the LC3 II levels were already at their maximum, and no further increase was observed even with DH treatment (Fig. 5c). Additionally, these findings were replicated in normal MuSCs (Fig. 5d).

Furthermore, to ascertain whether the autophagy marker LC3 is modulated through a mammalian target of rapamycin (mTOR)-dependent or mTOR-independent pathway, Torin, an mTOR inhibitor, was administered to cells. In the absence of amino acids, an increase in LC3 upon DH treatment was noted, which was attributed to DPH but not to 8-CT (Fig. 5e). Remarkably, this DPH-induced phenomenon persisted even under amino acid-depleted conditions (Fig. 5e). This pattern of LC3 protein expression was further substantiated through a cell immunofluorescence experiment conducted on C2C12 cells. Here, an increase in fluorescence intensity was detected after DPH and DH treatment (Fig. 5f, g). In summary, DH augments muscle proliferation and enhances muscle differentiation through DPH.

### Diphenhydramine in dimenhydrinate induces autophagy through an mTOR-independent pathway

**Fig. 6:**
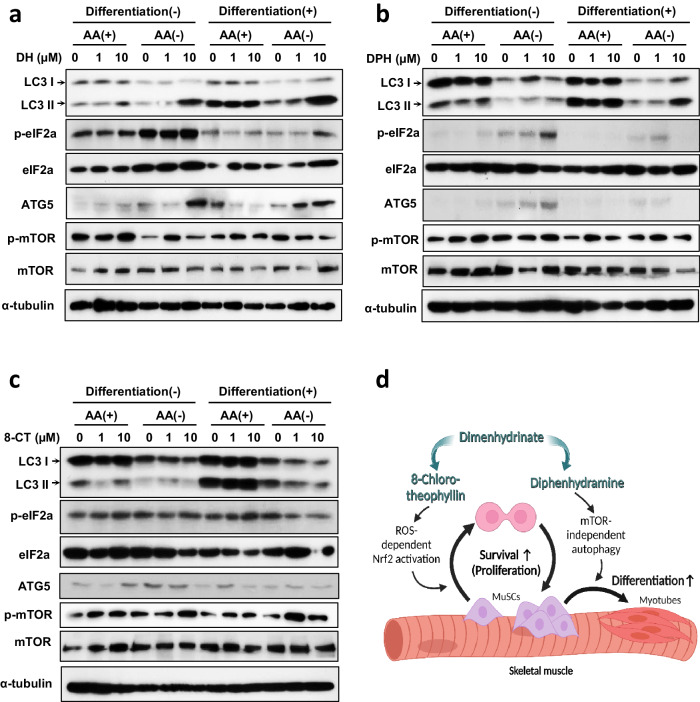
Diphenhydramine in dimenhydrinate induces autophagy via mTOR-independent LC3 accumulation. Immunoblotting for mTOR-independent LC3: **a** DH; **b** DPH; and **c** 8-CT. Cell differentiation was conducted over 24 h, and the media was exchanged with or without amino acids for another 24 h. **d** Schematic representation of the muscle regeneration mechanism mediated by DH and its components. DH dimenhydrinate, 8-CT 8-chlorotheophylline, DPH diphenhydramine.

In our initial research, an increase in LC3 levels, induced by DH, was observed even when mTOR signaling was suppressed (Fig. 5e). Consequently, we hypothesized that muscle differentiation induced by the upregulation of LC3 through DH operates independently of the mTOR pathway. Therefore, several pathways associated with LC3 under various conditions were analyzed, namely, the presence or absence of differentiation signals in muscle cells and harsh nutrient-deprived conditions versus nutrient-sufficient conditions. As expected, LC3 expression increased more robustly after differentiation than before differentiation, and consistent with previous results, DH triggered a dose-dependent increase in LC3 and autophagy-related 5 (ATG5) expression. Interestingly, the basal expression of LC3 was slightly higher under differentiated conditions than under control conditions, and surges in LC3 and ATG5 were detected mainly under amino acid-depleted conditions (Fig. 6a). DH did not appear to influence mTOR or its downstream signals. However, an increase in the phosphorylation of EIF2A, which halts protein translation and initiates mTOR-independent autophagy, was observed.

To determine the component within the DH that is responsible for inducing autophagy and the subsequent regenerative effect on muscle cells, 8-CT and DPH were used to treat cells under the same conditions, as illustrated in Fig. 6a. Intriguingly, an expression pattern similar to that generated by DH treatment was found in DPH-treated cells (Fig. 6b). Specifically, the upregulation of LC3 followed a similar pattern. In contrast, 8-CT was found to downregulate LC3 expression (Fig. 6c). In conclusion, DH promotes muscle regeneration through the antioxidative effect of 8-CT and fosters muscle differentiation through the induction of autophagy by DPH (Fig. 6d).

### Dimenhydrinate increases muscle regeneration in a toxin-induced mouse model

**Fig. 7:**
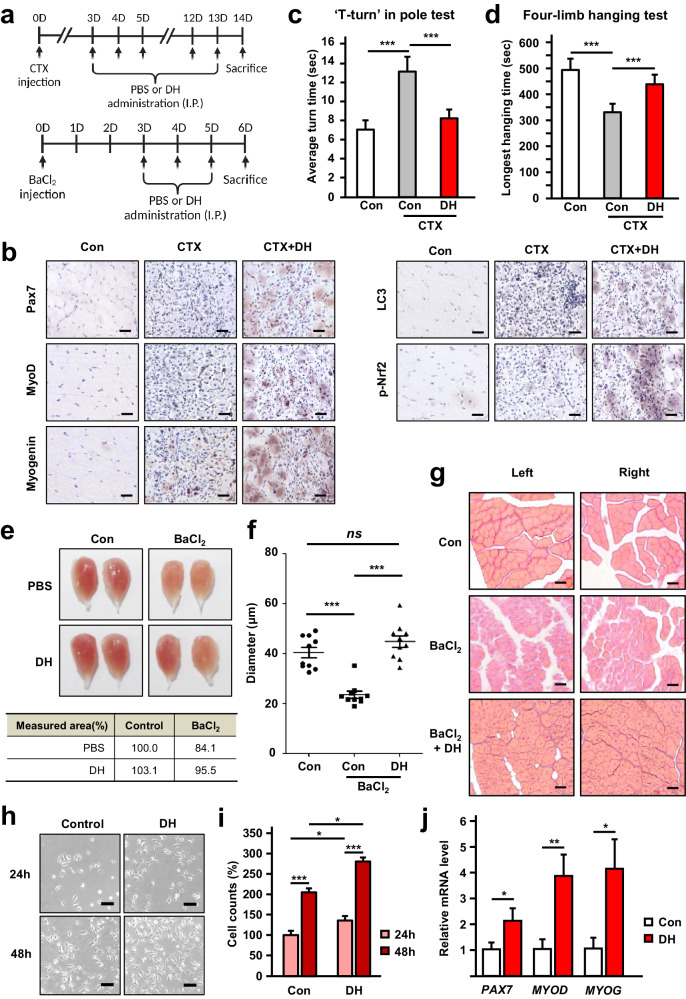
Dimenhydrinate exhibits muscle regeneration capability in a toxin-induced muscle damage model. **a** Schematic of the muscle damage model induced by cardiotoxin (CTX, 1 μg) or BaCl_2_ (1.2% w/v). PBS or 30 mpk DH was administered to each mouse by intraperitoneal injection. **b** Representative immunohistochemistry image of gastrocnemius muscle sections from the CTX-induced model. Pax7 was used as a marker of MuSCs, while MyoD and myogenin were used as markers of muscle differentiation. Scale bars = 100 μm. **c**, **d** Locomotor test of CTX-treated PBS- or DH-treated mice. **c** Time (s) taken for the “T-turn” in the pole; *n* = 3 for each group. **d** Longest time (s) spent hanging wire with four limbs. The maximum time was set at 600 s. *n* = 3 for each group. **e** Representative image of the gastrocnemius muscle in BaCl_2_-induced muscle damage model mice. The measured area was calculated using ImageJ. **f** Comparison of fiber diameter in BaCl_2_-induced muscle damage model mice with or without DH administration; *n* = 5 for each group. **g** Representative hematoxylin and eosin (H&E) staining image of the BaCl_2_-induced model. Scale bars = 200 μm. **h**–**j** Muscle satellite cells were isolated from 8-week-old MDX mice. The cells were incubated in differentiation media for 1 day with or without DH. **h** Representative microscopy image of primary myoblasts isolated from MDX mice with or without DH (10 μM). **i** Relative number of myoblasts isolated from MDX mice with or without DH (10 μM). **j** Relative mRNA expression of *PAX7*, *MYOD*, and *MYOG*. *n* = 3 for each group. ns not significant, *p < 0.05, **p < 0.01, ***p < 0.005. Error bars: standard deviation (SD). DH dimenhydrinate, MuSC muscle satellite cell.

To investigate the effect of DH on muscle regeneration and differentiation, cardiotoxin (CTX) or BaCl_2_ was used to induce myonecrosis in the gastrocnemius muscles of mice. In the CTX experiment, DH was administered daily for 11 days, starting 3 days after the CTX injection. Subsequently, muscle specimens were harvested and subjected to analysis on day 14 (Fig. 7a, upper section). Within the BaCl_2_ experiment, DH was administered daily for a duration of 3 days, beginning 3 days postinjection. Muscle tissues were then collected and subjected to analysis on day 6 (Fig. 7a, lower section). In a short-term damage-induced model utilizing CTX (Supplementary Fig. 4a), the muscle structure collapsed in mice treated with CTX, but the TA muscle was restored in mice treated with DH; the morphology showed more compact patterns (Supplementary Fig. 4b). Subsequently, the diameter of the muscle was measured, and the diameter of the muscle in the DH-treated group was significantly greater than that in the control group (Supplementary Fig. 4c). In addition to muscle diameter, fiber size was measured in muscle tissue samples to validate muscle recovery. In the CTX-induced model, most of the muscle fibers were short, while in the muscles of the mice treated with DH, there was a greater distribution of larger fibers (Supplementary Fig. 4d). Furthermore, when we conducted immunohistochemistry (IHC) on sections of gastrocnemius muscle from both the PBS and long-term DH administration groups, we observed notable increases in the MuSC marker paired box 7 (Pax7) within the damaged region of the long-term DH administration group, and the muscle differentiation markers MyoD and myogenin were also upregulated (Fig. 7b). Additionally, LC3 and p-Nrf2 levels were markedly increased by DH, which supports our previous findings in vitro (Fig. 7b). Collectively, these findings affirm the involvement of DH in muscle regeneration by promoting the proliferation and differentiation of MuSCs in vivo.

To assess whether these molecular alterations are concomitant with improvements in mobility, an in vivo muscle strength evaluation was conducted. The results revealed that, in the pole test, the DH-treated group exhibited an ~40% reduction in the average time required for a “T-turn” compared to that of the PBS-treated group (Fig. 7c). Moreover, in the four-limb hanging test, the DH-treated group demonstrated an increase of ~25% in the longest hanging time compared to the PBS-treated group (Fig. 7d). Collectively, these findings provide compelling evidence that DH administration promotes muscle damage recovery and substantially enhances muscle strength.

For further validation, a BaCl_2_-induced myonecrosis model was used. A decrease in the volume of the gastrocnemius muscle in the BaCl_2_-treated group was detected; however, an increase was observed in the DH-treated group (Fig. 7e). To investigate the recovery of muscles damaged by BaCl_2_, the gastrocnemius muscles were sectioned, and the diameter was analyzed. The results showed that the gastrocnemius muscles of the BaCl_2_-treated group were approximately half the diameter of those of the normal group. In contrast, the DH-treated mice had a diameter similar to that of the normal mice (Fig. 7f). In addition, when histological analysis was conducted using hematoxylin and eosin staining, the BaCl_2_-treated muscle tissue exhibited substantial damage; however, the density and morphology of the DH-treated muscles were almost identical to those of the normal leg muscles (Fig. 7g).

We isolated primary myoblasts from the hindlimb skeletal muscles of 12-week-old MDX mice to establish an in vitro model of DMD. The cells were cultured for 2 days in the presence or absence of DH to evaluate the implications of DH for this MDX model. Interestingly, we noted an increase in the myoblast number in the DH-treated MDX mice compared to the control mice (Fig. 7h, i). Furthermore, DH induced the expression of the differentiation markers *MYOD* and *MYOG* and the proliferation marker *PAX7* in MuSCs from MDX mice (Fig. 7j and Supplementary Fig. 2d). These results indicate that DH enhances proliferation even within myogenic cells from dystrophic muscle. In conclusion, DH has been shown to demonstrate outstanding effects in promoting muscle regeneration and recovery in toxin-induced muscle damage models and dystrophic mouse models.

### Dimenhydrinate increases muscle regeneration in a cancer formation and 5-FU chemotherapy-induced muscle wasting model

**Fig. 8:**
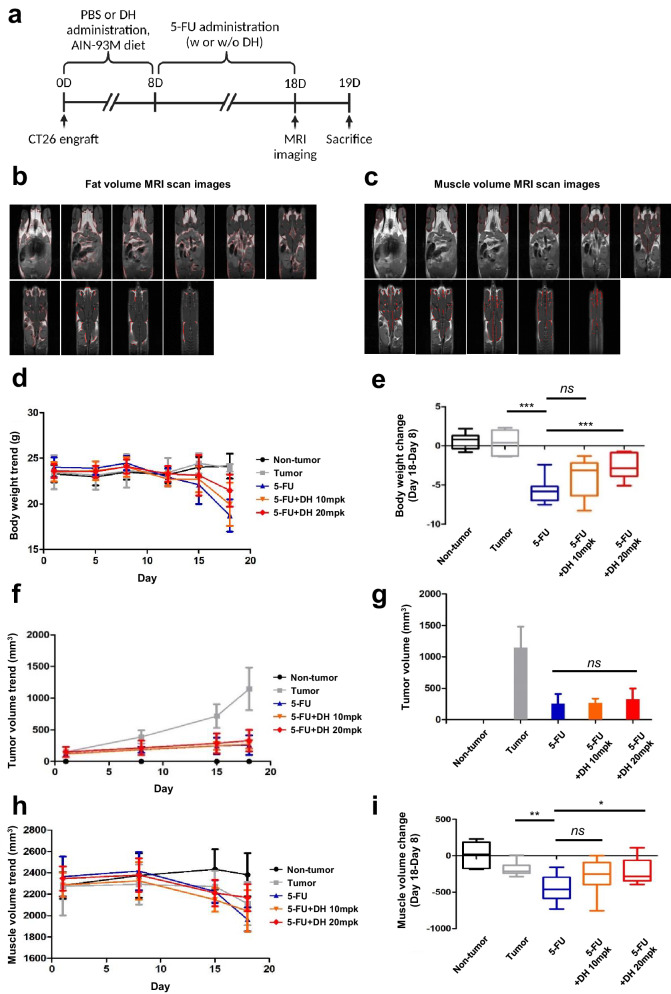
Dimenhydrinate has therapeutic efficacy against cancer and 5-FU chemotherapy-induced sarcopenia without promoting tumorigenesis. **a** Schematic representation of the overall schedule for assessing the therapeutic effect of DH (50 mpk) on cancer model mice and 5-FU chemotherapy-induced model mice. **b** Representative MR images for fat volume measurement (highlighted in red). **c** Representative MR images for muscle volume measurement (highlighted in red). **d** Graphical representation of body weight measurements for each group over time. **e** Relative change in body weight between day 18 and day 8 for each group. **f** Graph of tumor size measurements for each group over time. **g** Relative tumor volume for each group. **h** Graph of muscle volume measurements for each group over time. **i** Relative change in muscle volume between day 18 and day 8 in each group; *n* = 8. ns not significant, *p < 0.05, **p < 0.01, ***p < 0.005. Error bars: standard deviation (SD). DH dimenhydrinate.

In many case reports, sarcopenia can occur in cancer patients over the natural course of the disease^57^. To establish a cancer formation and 5-FU chemotherapy-induced sarcopenia model, we injected CT-26 colon cancer cells into BALB/c mice and treated them with DH. Moreover, we performed chemotherapy with 5-FU 8 days after tumor establishment (Fig. 8a). During the experiment, we monitored tumor size and body weight and measured fat volume (Fig. 8b) and muscle volume (Fig. 8c) using MRI (magnetic resonance imaging) (Fig. 8a). After chemotherapy with 5-FU, the body weight of the mice decreased sharply compared with that of the mice not treated with 5-FU (Supplementary Fig. 5a, b); however, when DH was administered, an increase in body weight was observed (Fig. 8d, e). This increase in body weight was not due to changes in tumor weight (Fig. 8f, g), indicating that DH does not affect tumorigenesis. Muscle volume was restored by DH treatment (Fig. 8h, i). By measuring fat volume changes via MRI, it was confirmed that fat reduction occurred due to chemotherapy. When DH was administered, the reduced fat content was also restored (Supplementary Fig. 6a, b).

## Discussion

Sarcopenia is highly prevalent not only in aging adults but also in individuals with certain fatal diseases, including progressive cancer and ALS^58–60^. However, little is known about the mechanism of muscle loss, and due to its diverse and complex etiology, there are currently no approved treatments^32^.

There is an urgent need to understand the mechanisms underlying the development of therapeutic interventions for sarcopenia, which requires further study. In response, we have introduced a unique approach called drug repositioning analysis using AI technology to develop sarcopenia therapeutics. Given that it is almost impossible to specify a particular causative gene for muscle loss, our approach was based on comparing the effects of drug treatment on gene expression and cellular processes, such as cell proliferation and differentiation, to predict the drug’s mechanisms of action. Consequently, utilizing the AI, we identified DH as a prospective candidate for sarcopenia therapy (Figs. 1 and 2).

DH is a mixture of two different compounds, 8-CT and DPH, and is known as a histamine-1 receptor antagonist used to prevent and treat motion sickness and nausea^61^. Initially, the main compound, 8-CT, is categorized as a stimulant drug of the xanthine class, and it is expected to have a physiological effect similar to that of caffeine, which is known for its ability to inhibit adenosine receptors^62^. However, little is known about its proliferative effect on muscle cells. The other compound, DPH, is an antihistamine drug with sedative effects. Thus, it can be prescribed to treat allergies, insomnia, and the common cold.

To verify whether DH discovered through AI technology positively affects muscle cell growth, an in vitro H_2_O_2_-induced stress model was constructed. Oxidative stress, induced by various factors, is a prevalent mechanism in cachexia and leads to elevated ROS levels^63^, oxidation-mediated protein modifications^64^, and impaired antioxidant defenses^48,65^. In line with the findings of recent studies exploring the link between muscle wasting and oxidative stress in cachexia^45–48^, we opted to simulate cachexia conditions in vitro by inducing oxidative stress through H_2_O_2_. DH treatment increased the proliferation of C2C12 mouse muscle precursor cells, despite treatment with H_2_O_2_-induced stress. 8-CT was responsible for the proliferative effect of DH (Fig. 4g, h). Specifically, when the cells were pretreated and subsequently exposed to H_2_O_2_, increases in p-Nrf2 levels were observed in both the 8-CT and DH groups (Fig. 4l). Nrf2 is crucial for safeguarding cells against oxidative stress. When Nrf2 is phosphorylated at serine 40 in response to ROS, it dissociates from Keap1 and translocates to the nucleus. Once in the nucleus, Nrf2 recognizes the antioxidant response element (ARE) and promotes the expression of ARE genes that encode antioxidant proteins, enabling cell survival and recovery from damage caused by ROS^66–68^. Therefore, we believe that 8-CT affects muscle cell proliferation by eliciting antioxidant effects through the activation of Nrf2 and enhancement of cell survival signals.

Along with ROS-induced oxidative stress, TNF-α suppresses the activation of Pax7, MyoD, and myogenin in myoblast cells^69,70^, indicating that this cytokine can disrupt the metabolism of myogenic cells at the early stage of differentiation, leading to delayed cell cycle exit and consequently impeding muscle repair^71–73^. Our findings suggest that DH successfully promoted cell proliferation under TNF-α-induced stress conditions. Specifically, 8-CT increased cell proliferation through the upregulation of cyclin-related genes and *PAX7* (Supplementary Fig. 3c–f), whereas DPH induced differentiation through the upregulation of *MYOD* and *MYOG* mRNA (Supplementary Fig. 3f). Moreover, it has been reported that ROS engage with specific cell signaling molecules in the TNF-α signaling pathway, including RIP, TRAF2, and JNK, to regulate cell survival and death^74,75^. This finding suggested that TNF-α signaling and oxidative stress are strongly related. Hence, subsequent comprehensive investigations are needed to determine the efficacy of these drugs in treating sarcopenia induced by TNF-α signaling and cytokines intricately linked with cancer.

We observed that DH promoted the differentiation and proliferation of muscle progenitor cells, and we confirmed that DPH promoted the differentiation of muscle cells. When muscle cells were under conditions similar to sarcopenia, such as amino acid depletion, DH treatment and DPH treatment were shown to increase the expression of LC3, an autophagy marker. When the cells were subjected to amino acid depletion, LC3 levels were increased in an mTOR-independent manner, indicating that autophagy activation occurred through the activation of eIF2 instead of mTOR (Fig. 6a–c). Autophagy is a process that degrades and recycles damaged proteins, organelles, and other cellular components to generate new energy sources for cell survival. Additionally, in a few recent studies, autophagy was identified as an essential process for inducing muscle differentiation^23,76^. Based on these results, DH can aid muscle regeneration and recovery, inducing autophagy in low-nutrient conditions. In addition, in muscle RNA-seq data obtained after DH administration in cancer formation and in a 5-FU chemotherapy-induced muscle wasting model, we found that DH promotes biological processes such as translation and peptide biosynthetic processes, which are thought to be related to autophagy (Fig. 3).

Given that sarcopenia is a symptom in various diseases, we employed three distinct mouse models, including cardiotoxin- (Fig. 7a–e), BaCl_2_ (Fig. 7f–h), and chemotherapy-induced sarcopenia models (Fig. 8) to mimic sarcopenia in mice and validate the therapeutic efficacy of DH. Several chemotherapeutic agents are postulated to directly modify host cell processes involved in protein metabolism^77^, and 5-FU was shown to accelerate skeletal muscle loss in mouse models^78,79^. To date, chemotherapy continues to be a central component of cancer treatment, and we have established a murine tumor and chemotherapy model that to validate the therapeutic efficacy of this drug. Our results demonstrated that DH was effective in alleviating the symptoms and associated consequences of muscle damage in all three models.

In conclusion, it is difficult to expect that a drug targeting a single gene associated with muscle loss would be effect in treating every individual with sarcopenia. However, based on the findings of the present study, DH may exert therapeutic or preventive effects on various muscle loss conditions, including rare muscular diseases, cancer-associated sarcopenia, and malnutrition. Therefore, DH may be considered a promising drug candidate with low toxicity for the treatment of sarcopenia.

### Supplementary information

Supplementary Information

## Data Availability

All of the data are available in the main text or the Supplementary Information.
