# Supplementary material for: Transcriptome-based deep learning analysis identifies drug candidates targeting protein synthesis and autophagy for the treatment of muscle wasting disorder
Source: Exp Mol Med. 2024 Apr 1;56(4):904–21. doi: 10.1038/s12276-024-01189-z (PMC11059359; doi:10.1038/s12276-024-01189-z)

Supplementary information

Transcriptome-based Deep Learning Analysis Identifies Drug Candidates Targeting Protein Synthesis and Autophagy for the Treatment of Muscle Wasting Disorder

Supplementary Figures

Supplementary Table 1: List of GEO databases a Comparison between cachexia patients or not. **b** Comparison between proliferative myoblast of not.

Supplementary Table 2: Top 100 Chemical list from GEO databases a The list from GSE20571. **b** The list from GSE34111**.** DH belongs into Top 100 chemical list both GEO databases. Abbreviation; DH: dimenhydrinate.

Supplementary Fig. 1: The difference of gene expression in the leg muscles between tumor-bearing mice and normal mice. a Tumor status drive global variation. Multidimensional scaling shows that dimension 1 captures nearly all variation related to tumor status in the overall sample population. Ellipses are centered on the group Euclidian center with distance from center showing the 80% confidence interval. **b** Volcano plot of control versus tumor-bearing samples. GO pathway over-representation in genes which are significantly upregulated in skeletal muscle of tumor-bearing versus control mice. **c** Cellular Components GO ontology. **d** Biological Processes GO ontology **e,f** GO pathway over-representation in genes which are significantly downregulated in skeletal muscle of tumor-bearing versus control mice. **e** Cellular Components GO ontology **f** Biological Processes GO ontology.

Supplementary Fig. 2: Dimenhydrinate promotes muscle satellite cell proliferation and differentiation. a-c Muscle satellite cells were isolated from 8-week-old wild-type mice: **a** Cell counting assay demonstrating the dose-dependent effects of DH under ROS-induced conditions. **b** Cell counting assay for DH, 8-CT and DPH under the ROS-induced conditions. **c** Relative mRNA expression of *PAX7*, *MYOD*, and *MYOG* under the ROS-induced conditions. **d** Immunoblot assay for myogenic markers, MyoD and myogenin of MuSCs from MDX mice. Differentiation was induced for 3 days. For supplementary Fig. a-c, H_2O_2 (400μM) was administered with or without chemicals for 24 h at a concentration of 10μM respectively. *ns*: not significant, *: $p<0.05$, **: $p<0.01$, ***: $p<0.005$. n=3. Error bars: Standard Deviation (SD). Abbreviations; DH: dimenhydrinate, CQ: chloroquine.

Supplementary Fig. 3: Dimenhydrinate ameliorates TNF-α induced stress by augmenting C2C12 cell proliferation and differentiation. a Cell counting assay demonstrating the dose-dependent effects of DH under TNF-α induced stress conditions (24h). **b** Immunoblotting of p21 following a 24 h treatment with or without TNF-α or DH. **c-e** Relative mRNA expression of cell cycle-associated markers: **c** *CCNA1*, **d** *CCNE1*, **e** *CCND1*. **f** Relative mRNA expression of *PAX7*, *MYOD* and *MYOG*. All chemicals were added at a concentration of 10μM respectively, rhTNF-α at a concentration of 40ng/ml. *ns*: not significant, *: $p<0.05$, **: $p<0.01$, ***: $p<0.005$. Error bars: Standard Deviation (SD). Abbreviations; DH: dimenhydrinate, 8-CT: 8-chlorotheophylline, DPH: diphenhydramine.

Supplementary Fig. 4: The short-term administration of dimenhydrinate in a CTX-induced mice model facilitates the recovery of muscle damage. a Schematic of the muscle damage model induced by CTX (1μg) and short-term administration of DH. **b** Representative hematoxylin and eosin-stained image from TA muscle section of CTX-induced model. Scale bars=50μm. **c** Comparison of fiber diameter with or without DH administration in CTX-induced mice. **d** Distribution of fiber size with or without DH in CTX-induced mice. *ns*: not significant, *: $p<0.05$, **: $p<0.01$, ***: $p<0.005$. Error bars: Standard Deviation (SD). Abbreviations; CTX: cardiotoxin, DH: dimenhydrinate, TA: tibialis anterior.

Supplementary Fig. 5: Representative MRI image between 5-FU treated mice or not. a Representative MRI image of coronal angle. **b** Representative MRI image of axial angle. The

part marked with a red line represents the volume of fat.

Supplementary Fig. 6: Dimenhydrinate displays therapeutic efficacy in a cancer-induced cachexia model without promoting tumorigenesis a Graph of fat volume measurements for each group over time. b Relative change in muscle volume between day 18 and day 8 for each group. *ns*: not significant, *: p<0.05, **: p<0.01, ***: p<0.005. n=8. Error bars: Standard Deviation (SD). Abbreviation; DH: dimenhydrinate.

Supplementary Table 1

a

Cohorts	GEO No.	Control (sample num.)	Sarcopenia (Sample num.)
1	GSE20571	Weight stable control (14)	Cachexia (13)
3	GSE34111	Healthy control (6)	Cancer cachexia (12)

b

Cohorts	GEO No.	G0 arrest (sample num.)	proliferation (Sample num.)
1	GSE38769	Non-proliferative myoblast (2)	Proliferative myoblast (4)

Supplementary Table 2

a GSE20571

4,5-dianilinophthalimide	hycanthone
5182598	idazoxan
5224221	idoxuridine
5248896	imipramine
5252917	iobenguane
5255229	isosorbide
5279552	levocabastine
AG-012559	lincomycin
AG-028671	liothyronine
AH-23848	lysergol
amiloride	melatonin
aminohippuric acid	mesoridazine
amodiaquine	mestranol
atropine methonitrate	methantheliniumbromide
benfluorex	methocarbamol
benzylpenicillin	MG-262
beta-escin	mianserin
bezafibrate	nalbuphine
blebbistatin	napelline
calcium pantothenate	naringin
carcinine	nifedipine
carmustine	nifuroxazide
chlorambucil	nimesulide
cicloheximide	ouabain
cinchonidine	oxedrine
clenbuterol	oxprenolol
clindamycin	PHA-00816795
convolamine	phenazone
cortisone	pilocarpine
CP-64552501	prenylamine
dehydrocholic acid	Prestwick664
dextromethorphan	Prestwick920
diazoxide	pridinol
dimenhydrinate	propranolol
disulfiram	pseudopelletierine
dorzolamide	puromycin
doxylamine	securinine
emetine	serotonin
epiandrosterone	spiradoline
estriol	sulfadoxine
etacrynic acid	sulfaguanidine
etamivan	suloctidil
ethosuximide	thioguanosine
fasudil	tolazamide
fendiline	tomatidine
flurbiprofen	trimethoprim
furaltadone	tubocurarine chloride
gramine	xylazine
hecogenin	xylometazoline
hesperidin	Y-27632

b GSE34111

2-aminobenzenesulfonamide	josamycin
3-acetamidocoumarin	lasalocid
4-hydroxyphenazone	leflunomide
5224221	levobunolol
5707885	levomepromazine
adiphenine	lisuride
amitriptyline	lomustine
atractyloside	lovastatin
bacitracin	maprotiline
benzathine benzylpenicillin	merbromin
benzocaine	meteneprost
betulin	methyldopate
betulinic acid	metitepine
calcium folinate	MK-886
canadine	monensin
carbenoxolone	nadolol
carbimazole	naringenin
cefaclor	oxyphenbutazone
cefamandole	pheneticillin
cefoperazone	piretanide
cefoxitin	pirlindole
chlorogenic acid	pivmecillinam
clemastine	podophyllotoxin
cyclic adenosine monophosphate	Prestwick-1103
cyclobenzaprine	Prestwick-642
diloxanide	Prestwick-675
dimenhydrinate	Prestwick-692
diphemanil metilsulfate	Prestwick-857
diphenhydramine	Prestwick-972
docosahexaenoic acid ethyl ester	pyrithyldione
econazole	quinpirole
erastin	semustine
etynodiol	spaglumic acid
felbinac	STOCK1N-35874
fludrocortisone	streptozocin
folic acid	sulfamonomethoxine
furosemide	suloctidil
gentamicin	suprofen
Gly-His-Lys	thapsigargin
guanabenz	thioperamide
harpagoside	tiletamine
heptaminol	timolol
ikarugamycin	tocainide
imatinib	trapidil
iohexol	trimethobenzamide
iopromide	vancomycin
iproniazid	vinblastine
isoflupredone	viomycin
isometheptene	W-13
isoxicam	withaferin A

Supplementary Fig 1

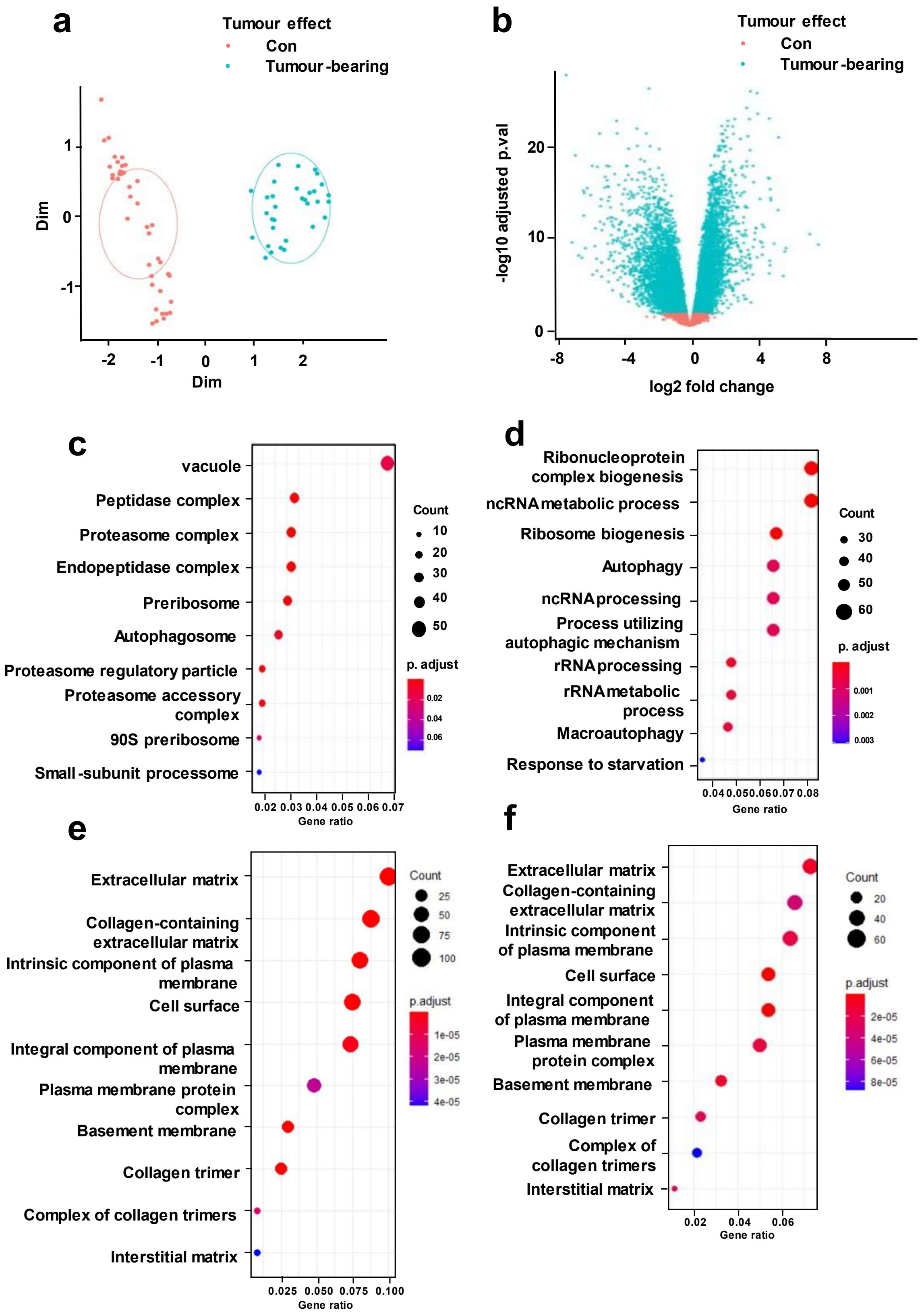

Supplementary Fig 2

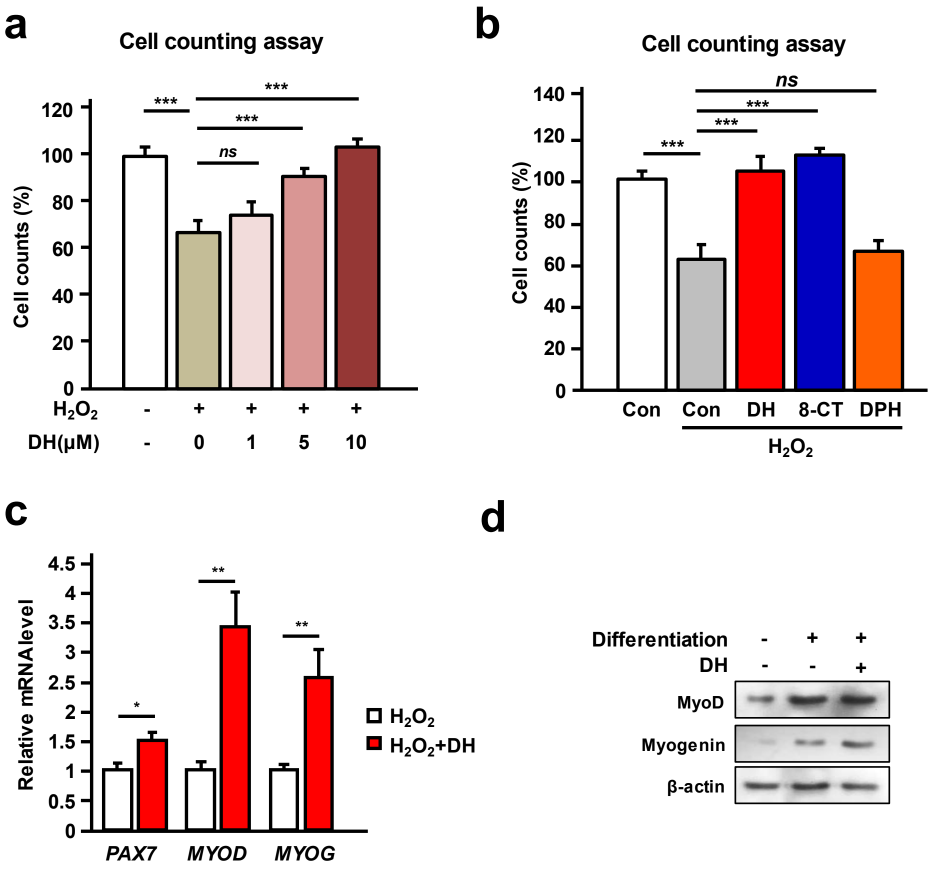

Supplementary Fig 3

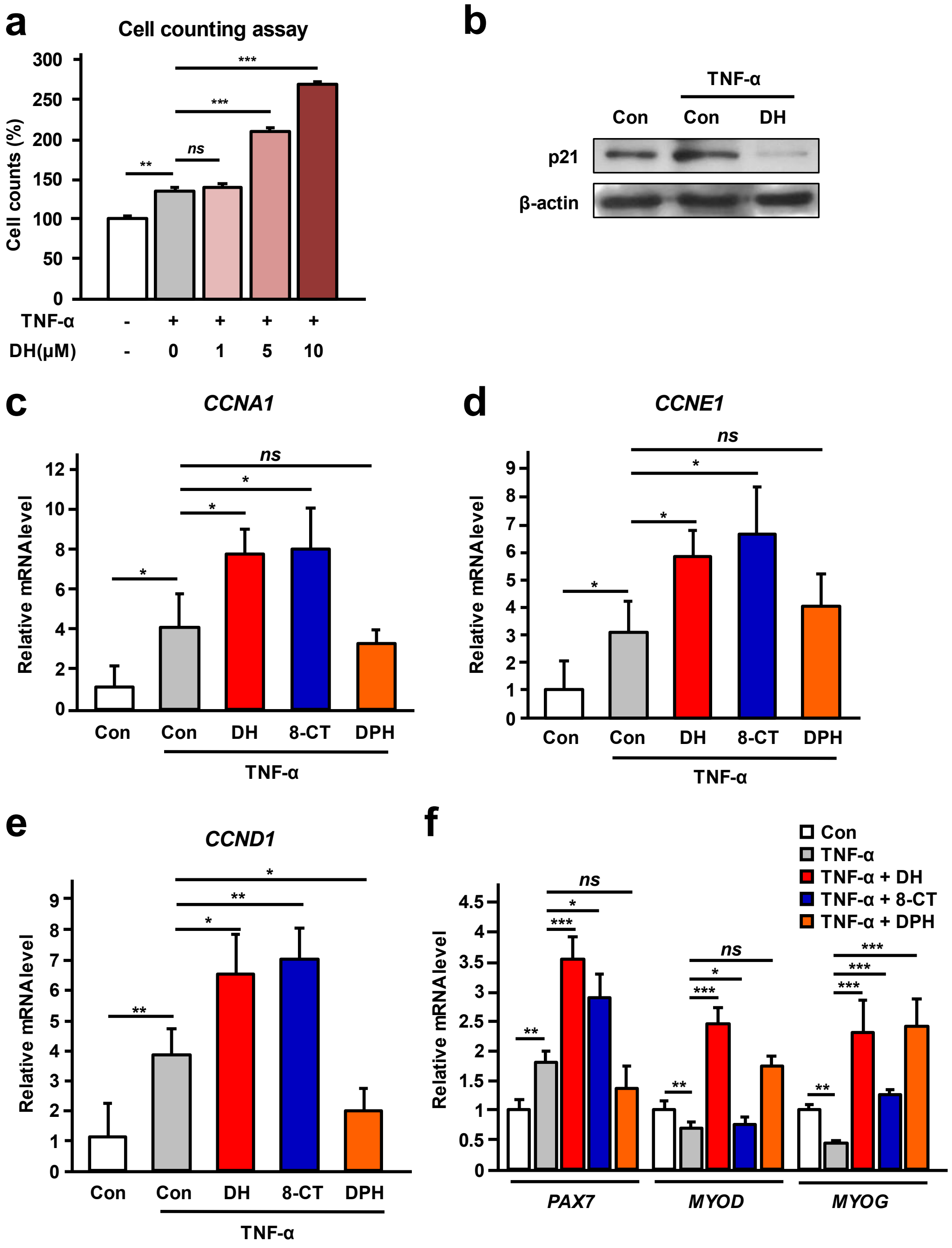

Supplementary Fig 4

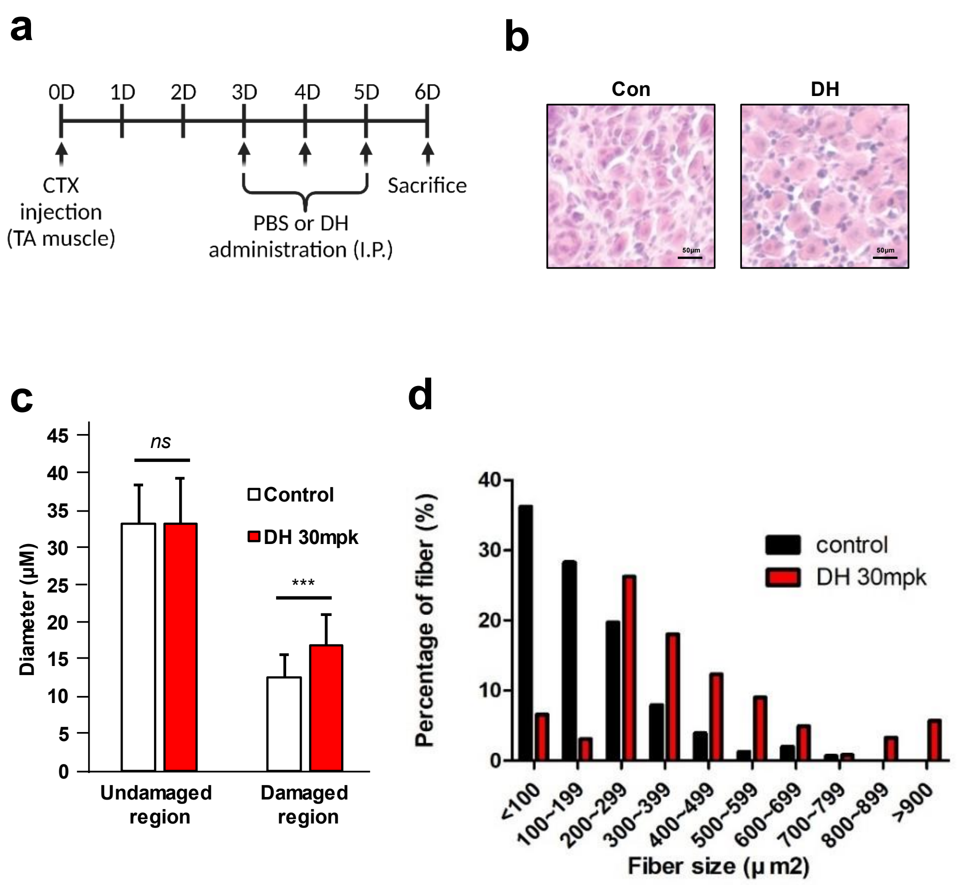

Supplementary Fig 5

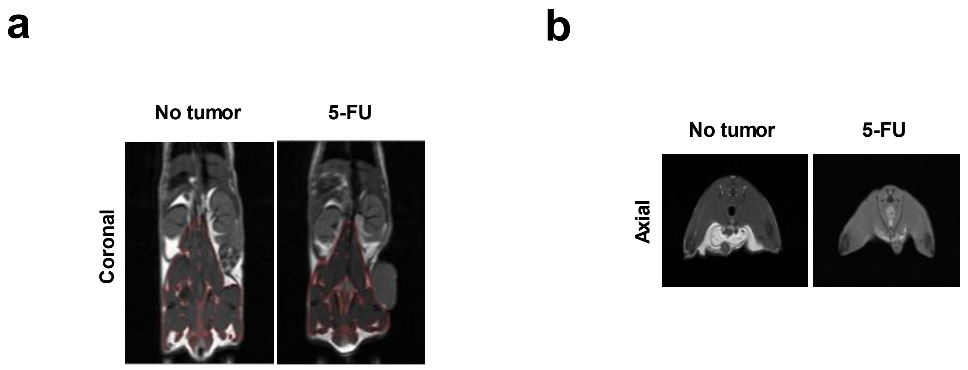

Supplementary Fig 6

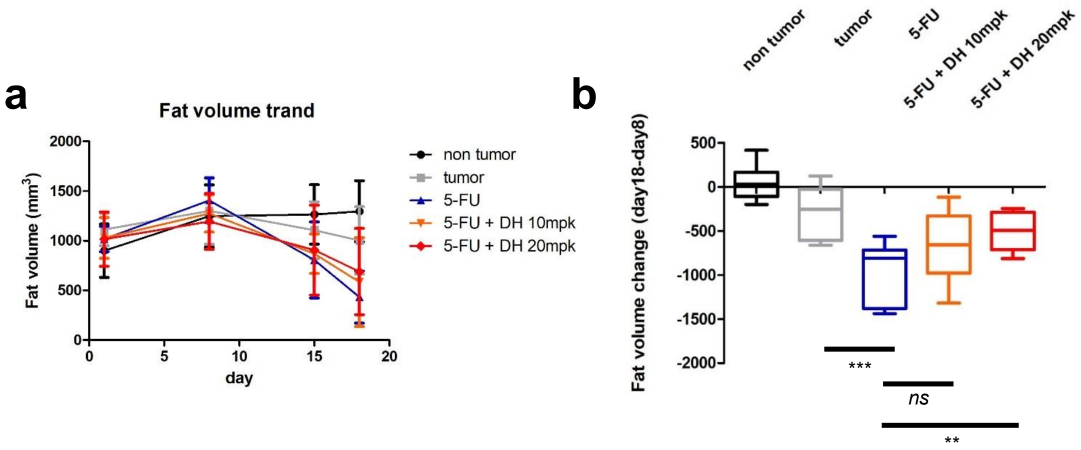

Supplement: Supplementary file 1 — Supplementary Information [file 12276_2024_1189_MOESM1_ESM.pdf]
